# A genome-wide screen for noncoding elements important in primate evolution

**DOI:** 10.1186/1471-2148-8-17

**Published:** 2008-01-23

**Authors:** Eliot C Bush, Bruce T Lahn

**Affiliations:** 1 Department of Human Genetics and Howard Hughes Medical Institute, University of Chicago, Chicago, Illinois, USA; 2 Department of Biology, Harvey Mudd College, Claremont, California, USA

## Abstract

**Background:**

A major goal in the study of human evolution is to identify key genetic changes which occurred over the course of primate evolution. According to one school of thought, many such changes are likely to be found in noncoding sequence. An approach to identifying these involves comparing multiple genomes to identify conserved regions with an accelerated substitution rate in a particular lineage. Such acceleration could be the result of positive selection.

**Results:**

Here we develop a likelihood ratio test method to identify such regions. We apply it not only to the human terminal lineage, as has been done in previous studies, but also to a number of other branches in the primate tree. We present the top scoring elements, and compare our results with previous studies. We also present resequencing data from one particular element accelerated on the human lineage. These data indicate that the element lies in a region of low polymorphism in humans, consistent with the possibility of a recent selective sweep. They also show that the AT to GC bias for polymorphism in this region differs dramatically from that for substitutions.

**Conclusion:**

Our results suggest that screens of this type will be helpful in unraveling the complex set of changes which occurred during primate evolution.

## Background

Humans possess numerous behaviors absent in other animals. Such behavioral specializations reflect anatomical, physiological and ultimately genetic modifications which occurred during the course of primate evolution.

The nature of the underlying genetic changes has long interested scientists. According to one influential viewpoint, key genetic differences in primate evolution resulted from changes in the regulation of gene expression [1]. Until recently however, there have been few examples of functionally significant cis-regulatory changes in the human lineage. This is beginning to change with the recent identification of several such cases. These include changes which occurred in a cis regulatory element of the PDYN gene in the lineage leading to humans [2], and changes in cis-regulatory elements of the LCT gene which lead to adult lactase persistence in a number of human populations [3,4].

Whole genome sequencing efforts may now allow us to identify more such cases. Several recent studies have used genome-wide screens to identify noncoding elements which may have been subject to positive selection in human evolution [5-7]. The basic approach is to look for genomic regions where the human branch contains a surprisingly large number of substitutions-that is regions which have an accelerated substitution rate in the human lineage. Pollard et al. used a likelihood ratio test approach that compared the branch lengths for each element with what would be expected given a genome wide model for conserved elements rescaled to the conservation level of the given element [5,6]. Prabhakar et al. developed a different method which incorporated variation in the neutral rate among lineages and loci into its estimation of acceleration [7]. In both cases the main focus was the human terminal lineage after its divergence from chimpanzee.

Here we present a new likelihood ratio test method for identifying acceleration in noncoding elements. Our approach is designed to account for lineage specific variation in mutation rates. We apply it not only to the human terminal lineage, as was done in previous studies, but also to other branches of the primate tree [8]. We identify lists of elements which have been accelerated in these branches, and in addition we report a follow up resequencing study on one of the elements we identified. In it we find evidence suggestive of a recent selective sweep in the human lineage.

## Results and Discussion

**Figure 1:**
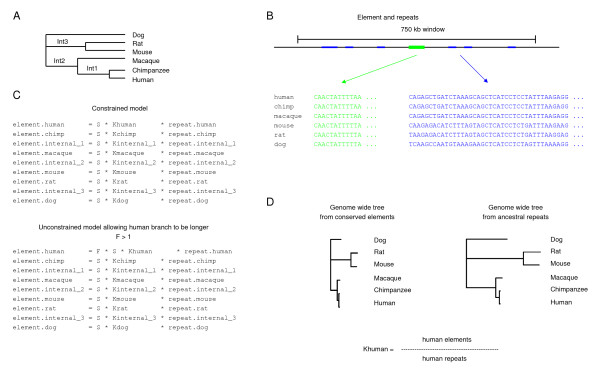
**Illustration of methods**. A. A phylogeny of the species we used with internal branches labeled. B. An element (green) and repeats (blue) within a 750 kb window. C. Constrained and unconstrained models. For each branch of the phylogeny, the element and the repeats are constrained to be proportional to each other. S is a tree wide scaling factor which accounts for the fact that elements vary in their general level of conservation (i.e. throughout the tree). The unconstrained model is the same as the constrained model, except that for whatever branch we are interested in (here the human terminal branch) we have added another variable F, which allows the element branch to be longer. We calculate the likelihood of the data under these two models. D. The constant of proportionality for each branch is based on the whole genome ratio between element and repeats for a particular branch.

We began with a genome wide set of conserved elements, the Phastcons elements [9]. We obtained these for the March 2006 release of the human genome and subjected them to a variety of filters to eliminate coding sequence, potential mis-alignments, and regions of low sequence quality (see Methods). We were left with 169,447 elements. These had a median length of 103 bp, and 1st and third quartiles of 73 and 161 bp respectively. For this set we obtained Multiz multiple alignments for 6 eutherian mammal species: human, chimpanzee, macaque, mouse, rat and dog [10]. We then used likelihood ratio tests to search these alignments for elements with an accelerated substitution rate in a particular lineage. Figure 1a gives an illustration of the phylogenetic tree for our species. In the description to follow we will refer to the various internal branches as they are labeled in Fig. 1a. We will call the human lineage after the divergence of chimpanzee the human terminal lineage, and the human lineage after the divergence of macaque (e.g the human terminal lineage plus internal branch 1) the long human lineage. In our description of our method below, we use the human terminal lineage as an example. However we applied this method also to the long human lineage and to internal branch 2.

Our goal was to identify elements which have undergone acceleration due to changes in selective pressure. To distinguish changes in selection from changes in mutation rate we compared the rate of molecular evolution in a given element with the rate in nearby ancestral repeats. For each element we examined, we obtained ancestral repeats in a 750 kb window surrounding (Figure 1b). Ancestral repeats represent sequence that is likely to be nearly neutral, and therefore allowed us to approximate the local mutation rate.

We then calculated the likelihood of the alignment for each element and its associated repeat alignments jointly under a constrained model, and then separately under an unconstrained model (Figure 1c and Methods). The unconstrained model is the same as the constrained with the addition of an extra variable which allows the human terminal branch to grow more rapidly. The likelihood ratio test (LRT) statistic is based on the ratio of these likelihoods, and will be larger in cases where the ratio between the human branch of the element and the human branch of the nearby repeats is significantly greater than the genome wide average. To assess the significance of these LRTs we used a nonparametric bootstrap method to calculate false discovery rates (FDR) for our data [11] (Methods).

Additional File 1 contains the 63 elements in our 10% FDR group for the human terminal lineage along with the trees calculated for each element, and for nearby repeats. (The file also contains the 10% FDR groups for for the long human lineage, and for internal branch 2.) These elements, which are generally very conserved among vertebrates, are changing extremely rapidly on the human terminal lineage. In all cases the estimated human branch length of the conserved element is greater than the local neutral branch length as estimated by our ancestral repeat tree. For the top 10 it is roughly an order of magnitude larger.

We wish to understand the genomic processes which produced these elements. To do so it is important to examine them carefully for biases in features such as their pattern of substitutions or physical positioning. Such biases can provide clues as to what forces produced them. We examined the position of our elements relative to Ensembl gene annotations and found that the accelerated group for the long human branch was enriched near gene deserts (defined as a region *> *500 kb without an Ensembl gene). 34% of our starting set of 169,447 elements fall in or adjacent to a gene desert. In comparison for the accelerated group on the long human branch, the proportion is 49% (Fisher's exact test p = 0.008181). For internal branch 2 and the terminal human branch this trend is not significant after we consider multiple testing.

In an earlier study, Pollard et al. found that in the 202 accelerated elements they identified on the human terminal branch there was a substitution bias from AT to GC. AT to GC substitutions constituted 57% of their total, while GC to AT were 29%. They suggested that biased gene conversion might contribute to this, an idea that was supported by the fact that their human accelerated elements were enriched in the terminal band of chromosomes. These regions tend to have a higher recombination rate, and are therefore a likely site of biased gene conversion [6]. It is interesting to ask if these patterns are also true for our group of accelerated elements. We counted AT to GC and GC to AT substitutions, omitting cases where the mutation occurred at a hypermutable CG dinucleotide. We found that in our human terminal group the bias to GC is weaker: 51% of substitutions are AT to GC while 31% are GC to AT. This ratio is not significantly different than what we find in the whole set of 169,447 elements which we screened. In the long human lineage group the proportion of AT to GC substitutions is 45%, which is less than that found in the whole set of elements. The same is true for the other primate lineage FDR groups. We should note that though we don't see a significant trend toward bias in the groups of elements we look at, certain individual elements do seem to show a strong trend. We will discuss one of these below. And we do find some tendency for our accelerated elements to be located in the final band of chromosomes. Our 10% FDR groups for the long human lineage, and for the terminal human lineage both have a higher proportion of elements in the final band of chromosomes than does the complete set of elements we screened (fisher exact test p = 0.005 and p = 0.15 respectively).

**Figure 2:**
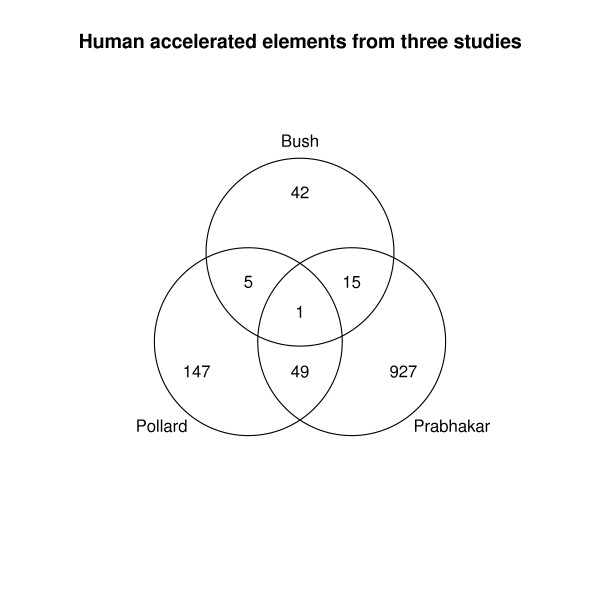
**Overlap with previous studies**. Venn diagram showing the overlap for human terminal branch elements from this study and two previous studies [6, 7, 39]. The group for the Pollard et al. study has and FDR adjusted p < 0.1, and the group for the Prabhakar study is p < 0.005.

The fact that our 10% FDR lists are not strongly biased toward GC is an indication that our method is selecting for a slightly different population of elements than is the method of Pollard et al. We examined the extent to which our results for the human terminal lineage overlap with those of their study, and another by Prabhakar et al. [6,7]. This is illustrated as a Venn diagram in Figure 2. It is striking that the results from the three studies have relatively little overlap. The majority of elements found by each study are unique to that study. This is true despite the fact that they used the same set of multiple alignments. These studies each have various methodological details which are unique. For example a feature unique to our study is the use of ancestral repeats to approximate the local mutation rate. The studies also vary in the level of conservation of the initial set of elements-Pollard et al. started with a smaller and more conserved set than we did. Clearly differences such as these have important effects. And clearly future refinements will be needed. The key to improving these screens will be studying their results in more detail, especially in the wet lab. This can provide independent evidence to help identify elements which have truly undergone positive selection.

In fact, screens of this type are by nature only a first step. They provide a list of candidate elements which can then be subjected to further experiments. This is so because various confounding factors can lead to spurious hits. For example, if an element becomes nonfunctional in the human lineage, this will lead to relaxation of constraint and a higher substitution rate. Accelerations of this type should be less dramatic than those caused by positive selection, but may nevertheless slip into our lists. The best way to think about these lists, is that they are likely to be enriched for cases of positive selection. But in any individual case, we must take the element and try to find additional evidence.

**Figure 3:**
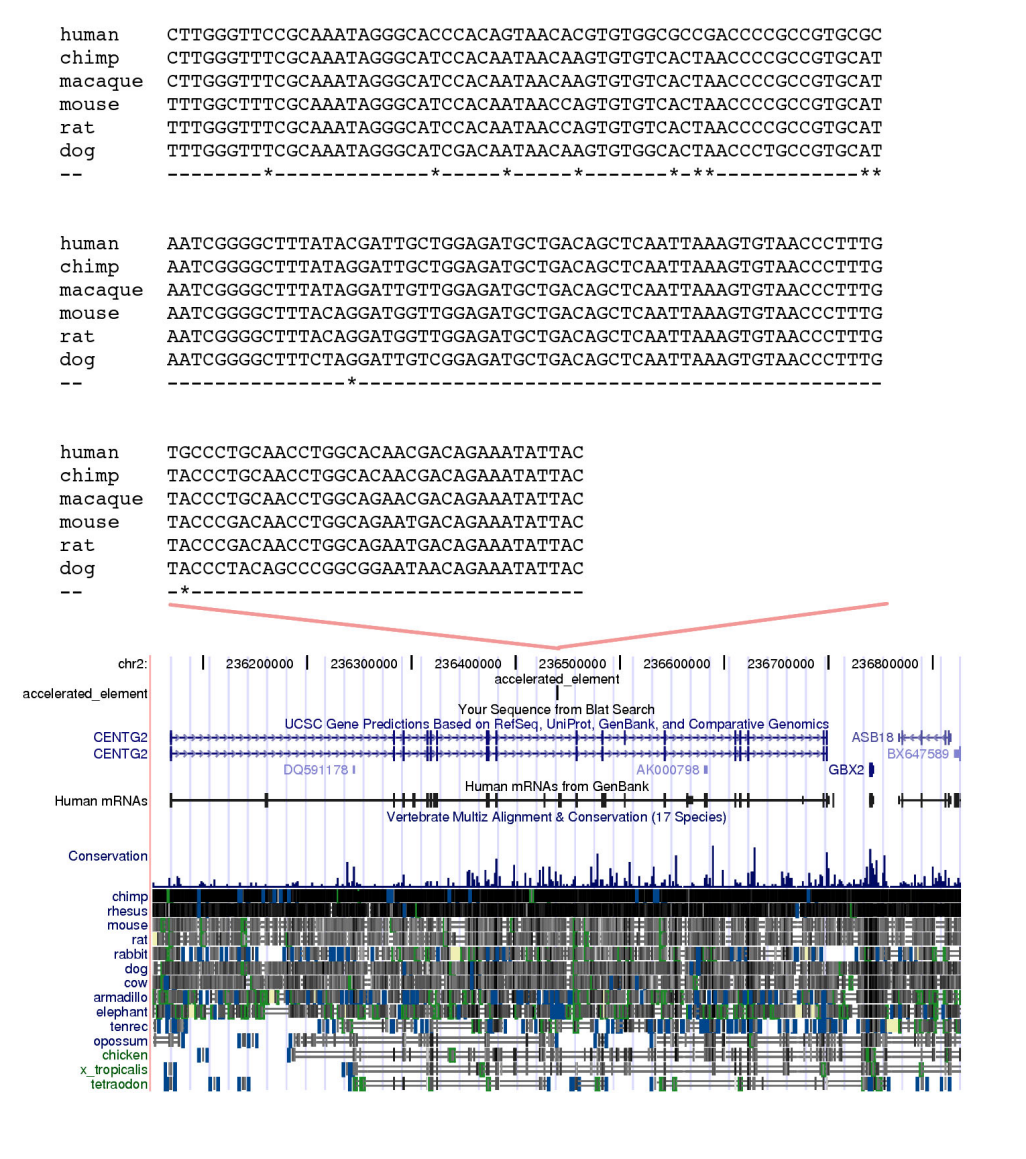
**An element on the human terminal branch**. Multiple alignment and genomic context for human terminal branch element 2 (HAR2 in Pollard et al. 2006). In the alignment * indicates columns where human is derived.

We now briefly examine several individual elements, with an emphasis on those near genes involved in brain development. The second element in the human terminal 10% FDR group is 155 bp long, conserved to Xenopus, and located at the distal end of the long arm of human chromosome 2. The ratio between its terminal human branch length (0.050) and the human branch of nearby ancestral repeats (0.0051) is about 10, which is dramatically larger than the genome wide average of 0.68. This difference is reflected in an LRT of 28.0, which is greater than any LRT in the nonparametric bootstrap set for this branch. This element also has the distinction of being the only one found in all three studies in Figure 2 (it is HAR2 in Pollard et al.'s naming scheme). It initially drew our attention because it is located near genes with interesting developmental and regulatory functions. It is in an intron of the centaurin gamma 2 gene (CENTG2), a brain expressed gene involved in membrane trafficking and the regulation of cGMP levels. CENTG2 belongs to a family of genes which have expanded greatly on the terminal human lineage [12]. This element is also about 300 kb downstream of gastrulation brain homeobox 2 (GBX2), a gene which is involved in midbrain-hindbrain segmentation in vertebrate embryos. Figure 3 shows an alignment and UCSC genome browser shot of this element. Using publicly available SNP data, Pollard et al. found low levels of polymorphism in the region around this element. Their results were suggestive of a selective sweep, but because it was based on SNP data with potential ascertainment biases, some questions remained.

To address this, we resequenced a 5.5 kb region around this element in a global human diversity panel of 90 individuals (Additional File 2). We found that the human specific substitutions in the element itself are fixed in our sample of humans. We also found that the surrounding region has a low level of polymorphism (pi = 0.00033), and a negative Tajima's D statistic suggesting an abundance of rare alleles (Tajima's D = -1.698, 0.05 < p < 0.10). Levels of polymorphism increase as one moves to the edges of the region we resequenced. In particular the 500 bp on the centromeric side of our region have a much higher level of polymorphism. This may be due to the fact that they overlap a recombination hotspot as defined by LDHot method [13,14] using Hapmap [15] and Perlegen [16] data. Excluding those 500 bp, pi in the remaining 5 kb region is 0.00017 and Tajima's D is -2.154 (p < 0.01). Pollard and colleagues also noted that the region of low polymorphism appears to be about 5 kb [6].

This polymorphism data is also interesting regarding the issue of AT to GC bias. Polymorphisms have been affected by forces such as selection and biased gene conversion only incompletely. As such, they are more reflective of mutation pressures than substitutions are [17]. In our resequencing data there is a very strong GC to AT bias (Table 1). This is strikingly different than the AT to GC bias in substitutions (fisher's exact test p = 1.101e-07). This says that in element 2, mutational forces are strongly tending to AT. However forces affecting substitutions, such as selection or biased gene conversion have driven in the other direction even more strongly. To us the fact that this difference is so dramatic suggests the involvement of selection, perhaps in addition to biased gene conversion. However we cannot eliminate other possibilities. Perhaps biased gene conversion is capable of producing such a dramatic difference by itself [18]. Or perhaps it was acting at this locus historically, but a recent change in recombination rate has reduced that influence. These caveats notwithstanding, we feel that overall our resequencing results are consistent with the idea that selection was involved in the evolution of human element 2. The region of reduced polymorphism we observe, while not large enough to be conclusive, is consistent with a selective sweep. One possibility would be positive selection for increased GC content in this region, perhaps related to the regulation of a nearby gene. As Pollard et al. suggested, multiple forces may be at work, including selection and biased gene conversion.

**Table 1:** GC vs. AT bias for polymorphisms and human lineage substitutions

	AT to GC	GC to AT	Neither
Polymorphisms	0.105 (2)	0.684 (13)	0.211 (4)
Substitutions	0.841 (37)	0.091 (4)	0.068 (3)

These data were taken from a 5.5 kb region around element 2. Counts are in parentheses. Cases where the mutation was at a hypermutable CG dinucleotide were ommited.

**Figure 4:**
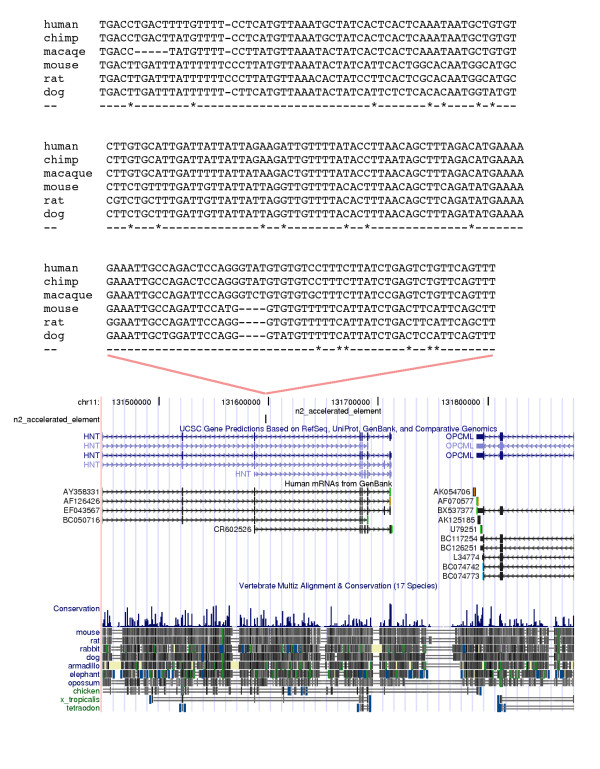
**An element on internal branch 2**. Multiple alignment and genomic context for the fourth element on internal branch 2. In the alignment * indicates columns where catarhines are derived.

As a second example, we consider an accelerated element on internal branch 2 (Figure 4). It is the 4th element in our 10% FDR group for this branch. It is 175 bp long and conserved to platypus. The ratio between the length of internal branch 2 in this element (0.17) and in nearby ancestral repeats (0.15) is 1.13, which is significantly larger than the genome wide average of 0.22 for this branch. This is reflected in the element's LRT of 32.6, which is greater than any LRT in our nonparametric bootstrap set for this branch. The element is located in an intron of the human neurotrimin (HNT) gene. HNT is an immunoglobulin domain containing cell adhesion molecule of the IgLON family which is expressed in the fetal brain and may be involved in controlling neurite outgrowth [19]. HNT is known to have several splice isoforms. Our element is located in an intron just after the transcription start of one of the isoforms, and could be involved in regulating the expression of that transcript.

For the future, we see several ways to improve our method. Ancestral repeats are known to have relatively less constraint than much of the genome. However it is worth noting that certain processes such as gene conversion may be more common in repeats, and that there may be variation between different kinds of repeats in terms of substitution rates. Such considerations may provide a basis for further improvements in our method. For example we could focus on certain categories of repeats which are known to have higher average substitution rates, or are less subject to gene conversion.

## Conclusion

We think it will be important to choose cases such as these and obtain direct experimental evidence about their function. One can begin by using transgenics to study expression in vivo. For example, in the case of the element located in the HNT gene we can attach the human and mouse versions to reporter constructs. We determine if these drive expression, and if so whether the human version's expression differs significantly from mouse. The hope is that in some cases the results of such work will suggest more detailed functional studies, specific to the biology of a particular adaptation.

We are optimistic that these lists and others like them will allow us to get a toehold in understanding the genetic basis of key adaptations in primate evolution. In this way, we can to begin to unravel the complex set of changes which have occurred in the course of primate evolution.

## Methods

We began with the Phastcons elements (based on the 17 way vertebrate mutliz alignments) [9,10] on the March 2006 release of the human genome. We subjected these to a number of filters before obtaining multiple alignments for six eutherian mammal species: human [20,21] (NCBI build 36.1 Mar. 2006), chimpanzee [22] (build 2 Mar. 2006), macaque [12] (v1.0 Jan. 2006), mouse [23] (build 36 Feb. 2006), rat [24] (v3.4 Nov. 2004) and dog [25] (May 2005).

We eliminated coding sequence using the UCSC genome informatics group's table browser [26]. This step was important because one category of spurious alignment is the case where a pseudogene has been aligned in one or more lineages. This situation could be mistaken for acceleration. We therefore filtered out all elements which overlapped UCSC Known genes, Refseq genes, Ensembl genes, mRNAs and spliced ESTs using the table browser on both the human and mouse genomes. We also eliminated elements which overlapped regions of low sequence quality (score<40) in any of our six genomes. We found that spurious alignments often involved cases where the aligning fragment in one of the species did not come from a syntenic genomic region relative to the aligning fragments in other species. We therefore applied a gene synteny filter. For each alignment, we examined the Ensembl genes nearby the aligning fragment in each species. To pass the filter, fragments needed to be less than three genes distant from a homologous gene in all six genomes. Another possible source of misalignments are cases where an element has undergone a recent duplication in one of the genomes. To eliminate this possibility, we filtered out elements which had multiple high blat hits in any of the six genomes. We also eliminated elements which were less than 50 bp long, or which were not present in all six of our species.

We obtained Multiz alignments for the 169,447 elements passing our filters using the six way mammalian synteny alignment available on the UCSC website [10]. In order to keep our molecular evolutionary models simple, we eliminated alignment columns with a CpG in any of the species. We also eliminated columns containing a gap.

By intersecting a genome wide set of repeats with the six way multiple alignments, we identified a set of repeats which were present in the ancestor of our six mammal species. Such elements are a good neutral proxy and we used them to estimate the local mutation rate around a given conserved element.

Our likelihood ratio test method is conceptually similar to a relative ratio test [27]. It is illustrated in Figure 1. For each element we obtained all ancestral repeats in a 750 kb window surrounding (Figure 1b). If an element had less than 6000 bp of repeats, we omitted it. For the whole set the median number of repeat bases per element was 17,930. We calculated the likelihood of each element and associated repeats jointly under a constrained model (weighting the element and repeat bases differently in the likelihood). We then did the same with the unconstrained model (Figure 1c). In the constrained model we require the rate of evolution of element and repeats to be proportional. The constant of proportionality for each branch is determined by the genome wide proportion (Figure 1d). To calculate it we determined the length of a particular branch for all elements taken together, and did the same for all repeats taken together. The ratio between these was the constant of proportionality for a given branch. It was necessary to calculate separate constants for each branch because these vary from branch to branch [22,28-31]. The constrained model also includes a tree wide scaling factor S, which accounts for the fact that different elements have different levels of conservation. Then for each branch we wish to test, we also obtain the likelihood for an unconstrained model. This model allows the element to evolve more quickly on that particular branch. From these likelihoods we calculate the likelihood ratio test (LRT) statistic for this element and this branch. We implemented this with the HYPHY package [32] using the HKY85 substitution model [33]. We are interested in cases where acceleration has occured in an individual element on a particular lineage. To isolate such effects, our models are designed to account for three other kinds of factors. Local variations in the mutation rate are accounted for by the fact that we are looking at the ratio between element and nearby reapeats. These should be affected equally by such variations. Lineage specific differences in constraint which are genome wide are accounted for by the constants of proportionality (e.g. Khuman, Kchimp in Fig. 1C.). And element to element variations in the (tree-wide) level of conservation are accounted for by S. differences between constrained and unconstrained models will reflect cases where the substitution rate in an individual element on a particular lineage has been accelerated.

To identify significant LRT values, we need to know the distribution of LRTs in elements which do not have a lineage and locus specific acceleration. We chose to do this with a nonparametric bootstrap method which involved creating pseudo elements by taking a random sample from all element alignment columns. We obtained mock ancestral repeats in the same manner. Both were size matched to actual data. The element and repeat sets produced by this method are a fair approximation of the null (no acceleration) condition. Such mock elements are unlikely to contain a cluster of accelerated columns. Such a cluster is what we would expect in a true accelerated element. The fact that they may contain any accelerated column by chance is conservative for our application. Contrained and unconstrained models were constructed in the same way as for real data. The constants of proportionality for these were created by calculating pseudo-element and pseudo-repeat branch lengths for the complete set of each taken together. We created 3,388,940 element repeat sets, and calculated an LRT for each. Using LRTs from these we calculated false discovery rate groups using the method of Benjamini and Hochberg [11].

The human samples and re-sequencing procedure are the same as in [34]. In this case we analyzed the data with phred, and phrap [35,36]. We called SNPs using polyphred with score set to 30 and confirmed them by eye using consed [37,38].

## Authors' contributions

ECB designed and implemented the study. ECB and BTL wrote the paper.

## Supplementary Material

Additional file 1 Tab delimited text file, containing the 10% FDR elements for the human terminal branch, the human long branch, and internal branch 2. Includes element position on the March 2006 human genome assembly, LRT score, and newick format trees for the element itself and nearby ancestral repeats. Click here for file

Additional file 2 Tab delimited text file containing our polymorphism data for a sample of 90 humans. Included are coordinates on the March 2006 release of the human genome as well as Coriell institute numbers for the samples. A "." indicates an ancestral genotype at a given position. Click here for file
